# Preoperative anxiety management in children. Benefits of humanoid robots: an experimental study

**DOI:** 10.3389/fsurg.2023.1322085

**Published:** 2023-12-08

**Authors:** Ilaria Franconi, Andrea Faragalli, Giulia Palego, Samuele Canonici, Ludovica Gatti, Alessandro Simonini, Edoardo Bindi, Giovanni Cobellis, Flavia Carle

**Affiliations:** ^1^ Operating Room, Salesi Children’s Hospital, AOU Ospedali Riuniti Ancona, Ancona, Italy; ^2^ Department of Biomedical Sciences and Public Health, Center of Epidemiology, Biostatistics and Medical Information Technology, Marche Polytechnic University, Ancona, Italy; ^3^ AOU delle Marche, Salesi Hospital Foundation Onlus, Ancona, Italy; ^4^ Pediatric Intensive Care Unit, Salesi Children’s Hospital, AOU Ospedali Riuniti Ancona, Ancona, Italy; ^5^ Pediatric Surgery Unit, Salesi Children’s Hospital, Ancona, Italy; ^6^ Department of Clinical Specialty Sciences and Odontostomatology, Università Politecnica of Marche, Ancona, Italy

**Keywords:** anxiety, children, humanoid robot, preoperative, pediatric surgery

## Abstract

**Objective:**

The purpose of this study was to determine whether the use of a humanoid robot (Estrabot) could reduce preoperative anxiety levels in children.

**Methods:**

An experimental study was conducted at Azienda Ospedaliero Universitaria delle Marche Hospital, involving the Pediatric Surgery ward and the Operating Room (OR). Patients aged between 2 and 14 years who underwent minor surgery were included. The Instruments used were the *Children's Emotional Manifestation Scale* to evaluate anxiety levels, and *Estrabot*, a humanoid robot that interacts with people. Medical records between April and May 2023 were analyzed and the data was anonymous. The level of anxiety is extrapolated in Pediatric Surgery during the administration of oral pre-medication, and in the Operating Room, during the induction of anesthesia. Patients were divided into an intervention group treated with Estrabot, and a control group without a robot.

**Results:**

The population consists of 60 patients (86.7% male) with a median (IQR) age of 6 (4–8) years. The median (IQR) anxiety score during premedication was 7 (5–11), while the median (IQR) anxiety score during anesthesia was 6 (5–10). A significantly lower level of anxiety was reported in the Estrabot group. Patients in the Estrabot group had significantly lower anxiety levels in different age groups.

**Conclusion:**

A humanoid robot can reduce preoperative anxiety levels in children during premedication and the induction of anesthesia.

## 1. Introduction

Surgery is a stressful experience for a child and his family, both from a physical and psychological point of view. It can become a traumatic event that leads the child to experience high levels of anxiety, mainly caused by the fear of being separated from their parents, the unfamiliar environment, and inadequate preoperative preparation. For these reasons, children appear tense, nervous, fearful, and agitated. The literature shows how preoperative anxiety can influence the intensity of postoperative pain and increase the induction time of anesthesia, which is known as the most stressful time for the child (1).

The management of these high levels of stress, to which the child and the family are subjected during the pre and post-operative period, represents a goal that arises in several different health professions. The nurse is certainly the most involved and suitable figure to assess and treat anxiety and to improve the preoperative experience of the patients, ensuring a quiet and reassuring hospital environment, and providing the child with the most suitable emotional support according to his degree of development (2, 3).

Non-pharmacological techniques and co-therapies have long been recognized as useful and valid tools for the control of fear and anxiety in a hospital setting, as well as for the management of procedural pain.

The American Academy of Pediatrics recommends a combination of pharmacological and non-pharmacological techniques to manage pediatric pain (4). To reduce the child's preoperative stress level, a good preoperative preparation of the young patient and their parents by the nursing staff is necessary. Furthermore, the presence of parents during the induction of anesthesia is essential. This does not make the anxious state disappear but it greatly reduces its impact on the duration of the induction itself (5). Other non-pharmacological methods have been studied with positive results, such as the use of natural sounds (6) and relaxation-guided imagery where the results obtained demonstrate the reduction of preoperative anxiety and post-operative pain (7).

The purpose of this study was to determine whether the use of a humanoid robot (Estrabot) could reduce preoperative anxiety levels in children.

## 2. Methods

### 2.1. Study design and setting

An experimental study was conducted at *Azienda Ospedaliero Universitaria delle Marche* Hospital, involving the Pediatric Surgery ward and the Operating Room (OR).

### 2.2. Sampling

• Inclusion criteria:

Patients aged between 2 and 14 years underwent minor surgery, such as surgery for inguinal hernia, circumcision, and orchidopexy.

• Exclusion criteria:

Patients under 2 years of age or above 14 years of age, who have undergone major surgery (appendicitis, peritonitis, intestinal obstruction, invagination, abdominal trauma, chest trauma, hemoperitoneum, hemothorax, liver fracture, kidney fracture, spleen fracture, ovarian torsion, testicular torsion, neuroblastomas, ovary neoplasms, congenital megacolon, anorectal malformations) or who have undergone urgency/emergency procedures.

### 2.3. Instruments

**Figure 1:**
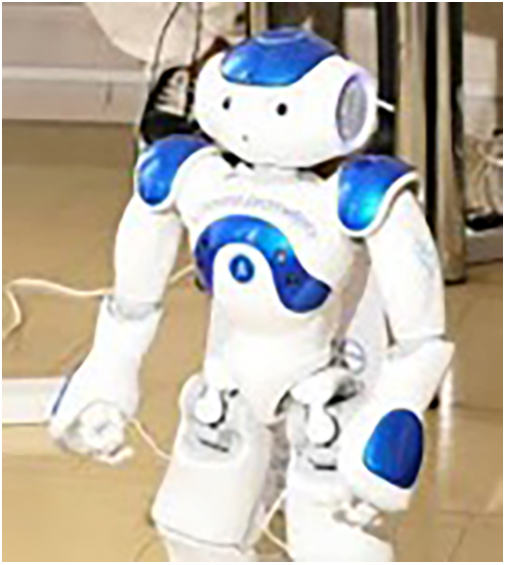
Estrabot: an humanoid robot that speaks in a child's voice. The model is NAO, developed by the French company Aldebaran Robotics in 2008 (acquired in 2015 by Japanese company Softbank), among the most widely used social robots in human-robot interaction, due to its affordability and broad functionality.

• Children’s Emotional Manifestation Scale (CEMS), which considers five variables: facial expression, vocalization, activity, interaction, and cooperation. For each of the five variables it is possible to assign a score from 1 to 5, therefore the overall score varies from 5 to 25. A lower score corresponds to a lower level of anxiety, compared to a higher score that indicates greater anxiety.
• Estrabot: a humanoid robot that speaks in a child’s voice. The model is NAO, developed by the French company Aldebaran Robotics in 2008 (acquired in 2015 by Japanese company Softbank), among the most widely used social robots in human-robot interaction, due to its affordability and broad functionality. NAO is 58 cm in height and weighs 5.6 kg; it has four directional microphones and speakers and two cameras which allow it to perform special features such as text-to-speech, speech recognition for 20 languages, object recognition, face detection and recognition, tracking, and more. Thanks to a complex system of joints, the entire body of the robot can move completely with 25 degrees of freedom: it can grab objects, move around, dance, and interact with people. A gyroscope, sensors, and a five-axis control unit provide balance during movements and exploration. It is fully programmable thanks to the included NAO Software Suite. Estrabot is the name chosen for the robot used for the activity. It has been purchased by the Salesi Foundation to implement projects in favor of children hospitalized at the Salesi Hospital in Ancona, Italy (Figure 1).

### 2.4. Data collection and analysis

The medical records were collected between April and May 2023. The level of anxiety is assessed in the Paediatric Surgery ward during the administration of oral premedication (sublingual midazolam), and in the Operating Room, during the induction of anesthesia. Patients were randomized into an intervention group treated with Estrabot, and a control group treated without the robot, according to the day of the week.

The experimental group is composed of children operated on Friday, who met the operator of Estrabot before the standard protocol (the same day or the day before), to allow them to know the project and tell something about their preferences, hobbies, and their favorite song. Using this information, the operator can make slight changes to the program, so that it can be easier for the robot to make every child feel safe and relaxed.

On the day of surgery the robot, accompanied by its operator, enters the child’s room with the nurse, explains to him in simple words the oral premedication procedure, and creates a first emotional contact by joking with him or, when possible, dancing or playing with the child and their parents.

It is then brought out of the room, before coming back when the child is accompanied to the lift which brings them to the surgery wing; here it reassures both the child and the parents and greets them. During this short journey, Estrabot plays the song chosen by the child, and when they reach the operating room it explains that before sleeping they will play inflating a balloon using a special mask; during the induction of anesthesia the robot pretends to blow in a similar mask, playing again the chosen song. As soon as the child falls asleep, both the robot and the operator quit the OR.

The control group is composed of children operated on Monday following the standard protocol without the company nor the explanations of the robot. This group of children is assisted by the ward nurse during the oral premedication, by auxiliary personnel during the journey to surgery, and by OR nurses during the induction of anesthesia. In both groups, the nurse who administered the premedication and one of those who assisted at the moment of anesthesia filled out the CEMS questionnaires during the procedures.

A non-parametric approach was followed. The characteristics of the investigated sample were summarized by absolute and percentage frequencies medians and interquartile range [IQR] for qualitative and quantitative variables respectively. To investigate the differences between groups, the non-parametric ANCOVA with smoothed regression and Young and Bowman test were applied. Two dependent variables were considered: the CEMS score evaluated during the administration of oral premedication and the CEMS score evaluated in the Operating Room during the induction of anesthesia; age was included as a covariate. One model for each dependent variable was applied. As a sensitivity analysis, the differences in CEMS scores between the two groups were stratified by 3 age classes (< 5 years, between 5 and 7 years, and >7 years) using the Mann–Whitney *U*-test. All statistical analyses were performed using R version 4.1.3.

## 3. Ethics

The Board of the teaching hospital evaluated the study. The research did not require Ethical Committee approbation because it does not involve direct medical, surgical, or pharmacological intervention on the patient. As suggested by the protocols of our hospital center, studies of this kind do not need approval by the Ethical Committee.

Study participation presents no known risks to the children and will not subject them to any additional pain or suffering. Children will receive standard medical management for their admission diagnosis.

Written informed consent to participate was obtained from all participants’ parents/guardians and oral one was obtained from all children.

## 4. Results

During the study period, a total of 60 patients were included in the group, 52 (86.7%) of them were males. The median (IQR) age was 6 (4–8) years. In the whole study population, the median (IQR) anxiety score during premedication was 7 (5–11), while the median (IQR) anxiety score during anesthesia was 6 (5–10). No difference in anxiety level was found between male and female patients, both during premedication: 7 (5–9) vs. 10 (8–16), *p* = 0.08, and during anesthesia: 6 (5–9) vs. 9 (5–14), *p* = 0.26, respectively.

Tables 1, 2 shows the comparison of anxiety levels during premedication and anesthesia between groups (Estrabot vs. Control) adjusted by age. A significantly lower level of anxiety was reported in the Estrabot group.

**Table 1:** Anxiety levels comparison between Estrabot and Control group: results of ANCOVA models.

		Estrabot (*n* = 30)	Control (*n* = 30)			
	Dependent variables	Adj. median^a^ (CI 95%)	Adj. median^a^ (CI 95%)^a^	RSE	Coefficient	*p*
CESM score:						
Model 1	*Premedication*	6 (5–9)	12 (10–13)	4.43	16.03	0.03
Model 2	*Anesthesia*	6 (5–8)	10 (8–11)	3.69	9.77	0.03

Each model was adjusted for age.

^a^ The adjusted median by smoothed regression model; CI 95%: 95% Confidence Interval;RSE: Residual Standard Error of the model.

**Table 2:** Anxiety levels comparison between Estrabot and Control group according to three age groups: results of Mann-Whitney *U*-test.

	Age <5 years			Age 5–7 years			Age >7 years		
	Estrabot (*n* = 11)	Control (*n* = 12)	*p*	Estrabot (*n* = 8)	Control (*n* = 12)	*p*	Estrabot (*n* = 11)	Control (*n* = 6)	*p*
Dependent Variables	Median (CI 95%)	Median (CI 95%)		Median (CI 95%)	Median (CI 95%)		Median (CI 95%)	Median (CI 95%)	
CESM score:									
*Premedication*	8 (5; 16)	13 (7; 20)	0,052	6 (5; 7)	8 (6; 13)	0,012	5 (5; 7)	9 (7; 23)	0,001
*Anesthesia*	6 (5; 13)	10 (8; 16)	0,033	5 (5; 10)	7 (5; 9)	0,143	5 (5; 6)	9 (5; 25)	0,008

## 5. Discussion

To our knowledge, this is the first experimental study on the use of a humanoid robot to reduce preoperative anxiety levels in children.

Various distraction strategies are useful in reducing preoperative anxiety. Playing comes naturally to children and is often their favorite activity. Providing an environment conducive to play activities, and toys, or using existing handheld game technology to make the environment less threatening has been shown to reduce anxiety (8), and enhance the cooperation of children with medical procedures (9) and anesthesia induction. Ensuring the presence of parents during anesthetic induction reduces anxiety before surgery in children (10). Other studies have demonstrated that non-pharmacological techniques, such as listening to music, may benefit preoperative anxiety (11). The use of a technologically enhanced device may effectively distract children and reduce their perceived anxiety. One recent study on 57 children has demonstrated the effectiveness of child–robot interaction for reducing pain and distress during vaccination (12). A second study has shown a reduction in distress for 40 pediatric oncology patients requiring central venous access (13). Logan et al. (14) proposed to use social robots as engaging tools to address the emotional needs of hospitalized children. The children exposed to an interactive teddy bear robot intervention showed more positive affect and they expressed greater levels of joyfulness and agreeableness than the other conditions. Our results show that a non-pharmacological intervention like a humanoid robot reduces anxiety in children during the pre-operative time and it might be an attractive solution to optimize perioperative care in children. As Feigal also stated in a study about non-pharmacological intervention for managing dental anxiety in children (15), behavior management needs to be flexible and individualized for each child; knowing in advance the child's preferences, the operator can adapt the Estrabot program to the patient's tastes, improving the humanoid robot's positive effect of the on the child's feelings.

A limitation of this study is the use of CEMS in adolescents, where more specific assessment scales (16, 17) should be used; CEMS was the tool routinely used in the study hospital.

Furthermore, we did not measure the anxiety level in children undergoing major surgery and in the parents. Future studies will be necessary to confirm our experimental results in children and adolescents using appropriate anxiety assessment tools and considering also major surgery, Parents’ anxiety status should be also analyzed. Therefore, the effectiveness of the humanoid robot and its determinants will have to be evaluated by performing observational studies in real clinical practice.

## 6. Conclusion

A humanoid robot can reduce preoperative anxiety levels in children during premedication and the induction of anesthesia.

## Data Availability

The raw data supporting the conclusions of this article will be made available by the authors, without undue reservation.
